# Establishing cut-off values on COPSOQ scales using depressive symptoms as criterion: a longitudinal study with a random sample of employees in Germany

**DOI:** 10.1007/s00420-026-02255-x

**Published:** 2026-11-10

**Authors:** Ruth Schäfers, Paul Maurice Conway, Hermann Burr, Uwe Rose

**Affiliations:** 1 Division 3 Work and Health, Federal Institute for Occupational Safety and Health (BAuA), Nöldnerstraße 40/42, Berlin, 10317 Germany; 2 Department of Psychology, University of Copenhagen, Øster Farimagsgade 2A, Copenhagen, 1353 Denmark

**Keywords:** Classification, Cohort study, PHQ-9, Psychosocial working conditions, Receiver operating characteristic, Workplace

## Abstract

**Objective:**

The association between psychosocial working conditions and depressive symptoms is well established. Going beyond the association, the present study aimed to identify optimal cut-off values for psychosocial working conditions using depressive symptoms as criterion.

**Methods:**

Data from the cohort study named Study on Mental Health at Work (S-MGA) were analysed using a representative sample of employees subject to social insurance at baseline and follow-up (2011/2012, *n* = 4,511; 2017, *n* = 2,637). Psychosocial working conditions were measured at baseline using eight scales from the Copenhagen Psychosocial Questionnaire (COPSOQ), all ranging from 0 to 4, while depressive symptoms were measured at follow-up using the Patient Health Questionnaire (PHQ-9; dichotomised). Cut-off values were determined by selecting the optimal combination of the corresponding sensitivity and 1 - specificity for each possible cut-off value. Receiver operating characteristic (ROC) curves illustrate the discriminatory power of the COPSOQ scales for classifying participants as cases with or without depressive symptoms.

**Results:**

The analyses revealed that the optimal cut-off values for the selected COPSOQ scales ranged from 1.38 to 3.50. Area under the curve (AUC) values ranged from 0.51 (95% CI 0.47–0.54) to 0.61 (95% CI 0.57–0.64).

**Conclusions:**

The method used allows to identify one optimal cut-off value on each COPSOQ scale. Due to the low discriminatory power of the COPSOQ scales for classifying individuals with depressive symptoms, the benefit of using the identified cut-off values as a classification tool is limited.

## Introduction

 While associations between psychosocial working conditions and depressive symptoms are well established (Madsen et al. 2017; Niedhammer et al. 2021; Rugulies et al. 2023; Theorell et al. 2015), there is still limited knowledge about optimal cut-off values for scales assessing psychosocial working conditions that could help identify employees with subsequent depressive symptoms. Diagnostic studies can address this gap by shifting the focus from examining psychosocial working conditions as risk factors for depressive symptoms to identifying optimal cut-off values on scales measuring psychosocial working conditions by using the presence or absence of depressive symptoms five years later as criterion. Thus, the key question addressed in the present study is which cut-off values perform best in terms of sensitivity and specificity for classifying individuals with subsequent depressive symptoms.

 To date, only a limited number of studies have investigated cut-off values that support the assessment of psychosocial working conditions based on established criteria. Most studies have focused on the psychometric evaluation of instruments used to assess workplace factors (e.g., Conway et al. 2018). These studies examined the ability of an instrument to discriminate between individuals or groups employing mental health indicators as external criteria. In occupational research, studies that aim to identify cut-off values capable of distinguishing individuals based on a binary mental health outcome and relying on representative samples from the German population are relatively rare (e.g., Diebig and Angerer 2021; Zeike et al. 2018). Of these, only one study has examined cut-off values for working conditions in relation to depressive symptoms (Diebig and Angerer 2021). The study by Diebig and Angerer (2021) addressed this issue by classifying participants according to the presence or absence of depressive symptoms (classification of depressive symptoms); however, it relied on a cross-sectional sample of 229 individuals. Studies identifying cut-off values applying this method while using a representative sample of employees and a longitudinal design are lacking. Therefore, the aim of this paper was to identify optimal cut-off values on scales from the Copenhagen Psychosocial Questionnaire (COPSOQ) for classifying individuals with subsequent depressive symptoms, using data from a cohort study based on a random sample of employees in Germany. We determined separate cut-off values for women and men due to gender-specific differences both in the prevalence of depressive symptoms (Otten et al. 2021; Salk et al. 2017) and in the relationship between working conditions and depressive symptoms (Demiral et al. 2022; Pohrt et al. 2022).

## Methods

### Sample

**Fig. 1:**
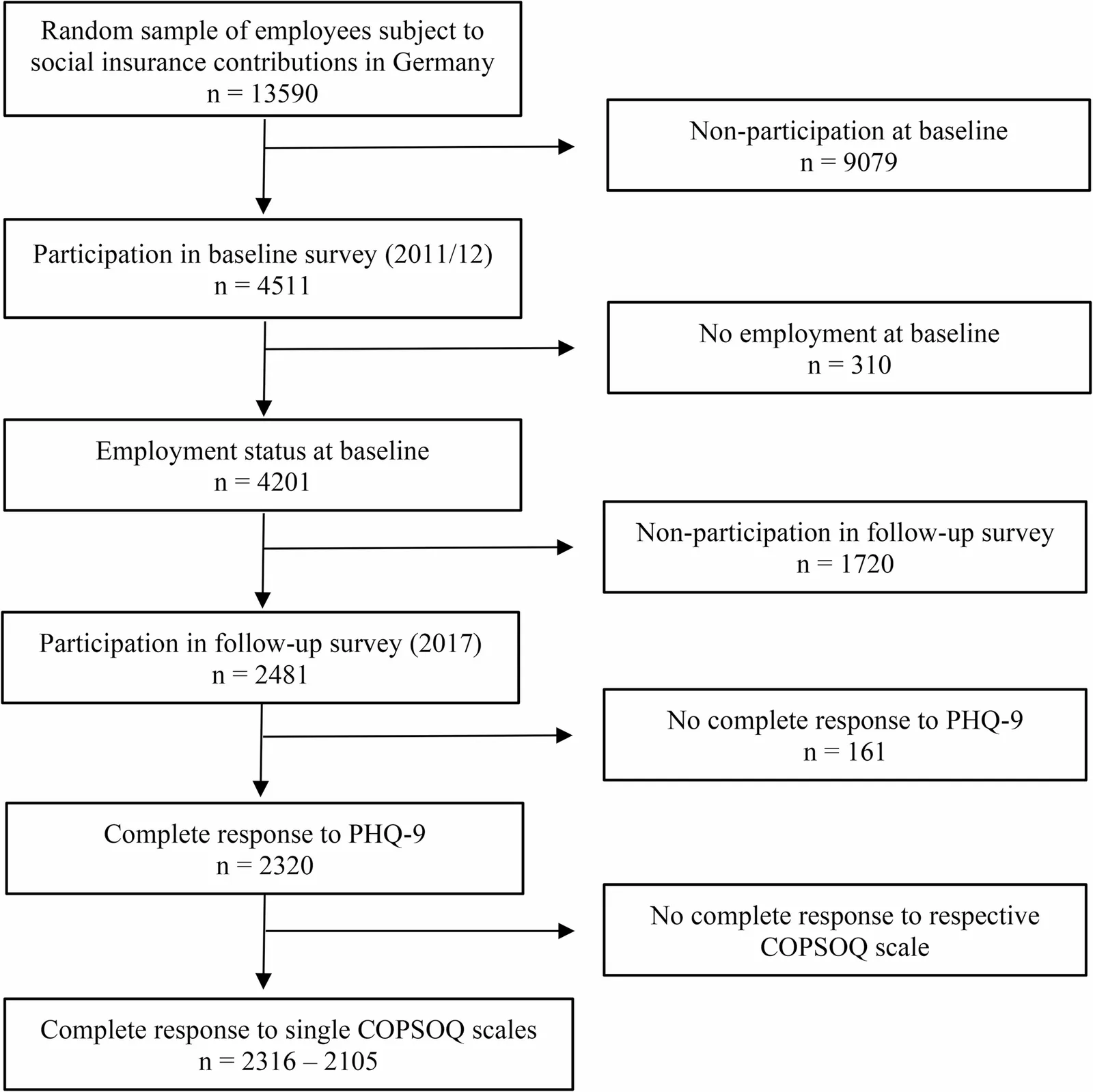
Flow chart from sampling to the analysis sample of the longitudinal study; Note: PHQ-9: Patient Health Questionnaire-9, a survey instrument for assessing depressive symptoms; COPSOQ: Copenhagen Psychosocial Questionnaire, a survey instrument for assessing various psychosocial working conditions

The present study was based on the Study on Mental Health at Work (S-MGA). Longitudinal data from a representative sample of employees subject to social insurance contributions in Germany (representing > 80% of the working population) were available at two waves of measurement (2011/12, *n* = 4,511; 2017, *n* = 2,637) (Rose et al. 2017). The sample comprised participants who were employed at both baseline and follow-up and had complete data for all included variables. Missing data were handled using listwise case exclusion (Lüdtke et al. 2007). The number of scales with complete information varied across the included psychosocial working conditions. To avoid loss of information, we formed eight analytical samples, one for each of the included scales (*n* = 2,105 to 2,316) (Fig. 1). All analyses were stratified by gender (men: *n* = 1,030 to 1,125; women: *n* = 1,075 to 1,191). More details on study design and sampling of the S-MGA study can be found in Rose et al. (2017).

### Variables

Psychosocial working conditions at baseline were assessed by eight scales from the Copenhagen Psychosocial Questionnaire (COPSOQ) (Nübling et al. 2006; Lincke et al. 2021). The eight scales measured job demands (i.e., work pace and amount of work) and job resources (i.e., influence at work, control over working time, possibilities for development, role clarity, social support from colleagues and quality of leadership). Each COPSOQ scale had values ranging from 0 (minimum) to 4 (maximum), which were calculated based on the mean of the component items, each measured using a five-point response scale. Depressive symptoms were measured at follow-up using the Patient Health Questionnaire (PHQ-9) (Gräfe et al. 2004). The PHQ-9 scores, ranging from 0 to 27, were calculated as the sum of the items included in the depressive symptoms PHQ-9 module and dichotomised at ≥ 5 (Kroenke et al. 2001). The decision to use a cut-off of ≥ 5 is justified by the fact that earlier stages of depressive symptoms are more prevalent in the sample of employees than in clinical samples, in which the higher cut-off of ≥ 10 is commonly used.

### Statistical analysis

Because non-parametric methods were used, no assumptions regarding the normality of the included variables were required. Accordingly, measures of central tendency and variability derived based on non-parametric methods are reported. For each potential cut-off value on each COPSOQ scale, the corresponding values for sensitivity (SENS) and specificity (SPEC) were calculated. As measures of diagnostic accuracy, SENS refers to the true positive rate, whereas SPEC refers to the true negative rate for the occurrence of an event. Receiver operating characteristic (ROC) analysis were performed to assess the diagnostic accuracy of the COPSOQ scales for the classification of depressive symptoms. For each of the eight COPSOQ scales, SENS and SPEC, as well as 1 - SENS and 1 - SPEC were considered in the determination of the optimal cut-off value. To select the optimal cut-off value, the maximum of the value pairs of 1 - SENS and 1 - SPEC was first determined for each possible cut-off value. The minimum of these maximum values was then identified, and the corresponding cut-off value was selected. Minimising the maximum error (min-max index) based on decision theory ensures that the sensitivity and specificity of a possible cut-off value are optimised simultaneously (Rose 1999).

The ROC curves were determined based on the SENS and 1 - SPEC value pairs for each possible cut-off value. The greater the discriminatory power of the scale, the closer the ROC curve is to the upper left-hand corner. If the classification is no better than chance, the ROC curve lies on the diagonal line (reference line), which corresponds to an area under the curve (AUC) of 0.5. The derived AUC and the corresponding 95% confidence intervals, as well as the p-value under the assumption that AUC equals 0.5, were calculated. The AUC indicates how accurately cases of depressive symptoms can be distinguished from non-cases based on the aforementioned adopted cut-off of ≥ 5 (Hajian-Tilaki 2013). According to Hosmer et al. (2013), AUC values between 0.5 and 0.7 are considered insufficiently discriminating. AUC values between 0.7 and 0.8 indicate acceptable discriminatory power and AUC values ≥ 0.8 indicate excellent discriminatory power.

## Results

Among the participants, 49% were men and 51% women. All participants had been born between 1951 and 1981. At baseline, the mean age was 47.37 years (SD 7.5 years).

The median PHQ-9 score was 3 for men (IQR = 4) and 4 for women (IQR = 5). The medians of the COPSOQ scales ranged from 1.75 to 3.33 among men and from 1.50 to 3.33 among women. Role clarity showed the highest values, whereas amount of work and influence at work showed the lowest.

The results of the statistical analysis stratified by gender are shown in Table 1. The main analysis indicated that, among men, the selected cut-off values ranged from 1.88 to 3.17. Among women, the cut-off values ranged from 1.63 to 3.50. The sensitivity for the optimal cut-off values was between 0.44 and 0.58 for men and between 0.43 and 0.70 for women, while the 1 - specificity was between 0.38 and 0.58 for men and between 0.35 and 0.64 for women. Values of the Min-Max index ranged from 0.46 to 0.58 for men and from 0.43 to 0.64 for women. The identified cut-off values were close to the reported medians of the COPSOQ scales.

**Table 1:** Gender-specific optimal cut-off values for each COPSOQ scale based on the Min-Max index and the values of the area under the curve (AUC)

Gender	COPSOQ scale	Cut-off value	SENS ^a^	1-SPEC ^b^	Min- Max ^c^	AUC ^d^	95% CI ^e^
men	Work Pace	2.50	0.58	0.58	0.58	0.51	0.47–0.54
	Amount of Work	1.88	0.54	0.44	0.46	0.57	0.53–0.60
	Influence at Work	1.88	0.56	0.48	0.48	0.56	0.53–0.60
	Control over Working Time	2.63	0.58	0.49	0.49	0.56	0.52–0.59
	Possibilities for Development	2.83	0.52	0.38	0.48	0.60	0.56–0.63
	Role Clarity	3.17	0.49	0.44	0.51	0.53	0.50–0.57
	Social Support from Colleagues	2.75	0.44	0.39	0.56	0.54	0.50–0.57
	Quality of Leadership	2.13	0.52	0.39	0.48	0.58	0.55–0.62
women	Work Pace	2.50	0.70	0.64	0.64	0.54	0.51–0.57
	Amount of Work	1.88	0.54	0.35	0.46	0.61	0.58–0.64
	Influence at Work	1.63	0.56	0.52	0.52	0.54	0.51–0.57
	Control over Working Time	2.13	0.59	0.48	0.48	0.57	0.54–0.60
	Possibilities for Development	2.83	0.50	0.41	0.50	0.55	0.52–0.58
	Role Clarity	3.50	0.64	0.51	0.51	0.58	0.55–0.61
	Social Support from Colleagues	2.75	0.43	0.37	0.57	0.56	0.52–0.59
	Quality of Leadership	2.38	0.57	0.41	0.43	0.61	0.57–0.64

^a^ SENS; sensitivity

^b^ 1-SPEC; 1-specificity

^c^ min-max index; minimisation of the maximum error

^d^ AUC; area under the ROC curve

^e^ CI; confidence interval

The ROC curves showed slight deviations from the diagonal reference line across all COPSOQ scales. Among men, AUC values ranged from 0.51 (95% CI 0.47–0.54, for work pace) to 0.60 (95% CI 0.56–0.63 for possibilities for development). Among women, AUC values ranged from 0.54 (95% CI 0.51–0.57 for work pace and influence at work) to 0.61 (95% CI 0.58–0.64 for amount of work; 95% CI 0.57–0.64 for quality of leadership). Thus, none of the AUC values indicated good discrimination according to the applied decision criterion (Hosmer et al. 2013).

## Discussion

In the present study, we identified cut-off values for a number of psychosocial working conditions using depressive symptoms as criterion. However, the identified cut-off values proved to have insufficient discriminatory power for distinguishing between cases with or without depressive symptoms. According to the evaluation criteria by Hosmer et al. (2013), all COPSOQ scales used showed rather weak associations with depressive symptoms. The deviations of the ROC curves from the reference line were minimal and the corresponding AUC values were low for all COPSOQ scales. Therefore, the present study suggests that using cut-off values on scales measuring psychosocial working conditions used in this study for identifying cases with or without depressive symptoms is not advisable for practice.

One strength of the current study is its reliance on a random sample of employees subject to social insurance contributions. This subgroup covers more than 80% of all employees in Germany, which allows for conclusions across a wide range of occupational groups and is therefore an advantage over previous studies (e.g., Diebig and Angerer 2021). In contrast to studies performed in clinical settings, this study enables the identification of cut-off values in a non-clinical, population-based setting. This allows for a preventive perspective on the potential development of depressive symptoms in the general population. Another strength is the use of the Min-Max index (Rose 1999) for selecting the cut-off value. If sensitivity and specificity are simply added together, as it occurs with the frequently used Youden index, low sensitivity can be compensated for by high specificity, or low specificity by high sensitivity. With the Min-Max index, the two errors are minimised simultaneously, which, in turn, leads to a unique solution compared with the Youden index.

As part of the non-parametric method, depressive symptoms at baseline were not included into the analysis. Consequently, although the identified cut-off values distinguish between individuals with and without depressive symptoms five years later, they should not be interpreted as thresholds for identifying individuals at risk of developing new depressive symptoms.

## Conclusion

In the present study, we focused on cut-off values for classifying individuals on a specific outcome, that is, depressive symptoms. The low discriminatory power of the identified cut-off values does not support their usability in the identification of cases with or without depressive symptoms. It is possible that COPSOQ scales would provide a better classification for outcomes that are considered more work related than depressive symptoms, such as long-term sickness absence, exit from work or limitations in work ability. Also, the use of different COPSOQ scales could lead to more reliable cut-off values. For example, emotional demands, unfair treatment or job insecurity have been shown to be better predictors of burnout symptoms (and subsequently depressive symptoms) than amount of work, possibilities for development, or the quality of leadership (Nübling et al. 2006; Lincke et al. 2021). Therefore, the present findings could be the result of the specific choice of demands and resources included in this study, meaning that the underlying idea of classifying individuals at risk of depressive symptoms based on cut-off values on instruments measuring psychosocial working conditions should be further explored in future studies.

## Data Availability

A scientific use file (SUF) containing both baseline and follow-up of the cohort is available at the Research Data Centre of the Federal Institute of Occupational Safety and Health (doi: https://doi.org/10.48697/smga.w1w2.suf.2).

## Abbreviations

AUC: Area under the Curve
BA: Federal Employment Agency
BAuA: Federal Institute for Occupational Safety and Health in Germany
CI: Confidence Interval
COPSOQ: Copenhagen Psychosocial Questionnaire
IAB: Institute for Employment Research
infas: Institute of Applied Social Sciences
IQR: Interquartile Range
PHQ-9: Patient Health Questionnaire
ROC: Receiver Operating Characteristic
SENS: Sensitivity
S-MGA: Study on Mental Health at Work
SPEC: Specificity
